# Correction: Prevalence and risk factors associated with rural women’s protected against tetanus in East Africa: Evidence from demographic and health surveys of ten East African countries

**DOI:** 10.1371/journal.pone.0315193

**Published:** 2024-12-04

**Authors:** Alebachew Taye Belay, Setegn Muche Fenta, Setegn Bayabil Agegn, Mitiku Wale Muluneh

The second author’s name is spelled incorrectly. The correct name is: Setegn Muche Fenta. The correct citation is: Belay AT, Fenta SM, Agegn SB, Muluneh MW (2022) Prevalence and risk factors associated with rural women’s protected against tetanus in East Africa: Evidence from demographic and health surveys of ten East African countries. PLOS ONE 17(3): e0265906. https://doi.org/10.1371/journal.pone.0265906.
