# Supplementary material for: Prevalence and risk factors associated with rural women’s protected against tetanus in East Africa: Evidence from demographic and health surveys of ten East African countries
Source: PLoS One. 2022 Mar 24;17(3):e0265906. doi: 10.1371/journal.pone.0265906 (PMC8947264; doi:10.1371/journal.pone.0265906)
Supplement: Attachment — Submitted filename: answer for plose 1 review.docx [file pone.0265906.s003.docx]

**Author’s Answer for Reviewer comments**

**Manuscript Title**: Prevalence and Risk Factors Associated with Rural Women’s Protected against Tetanus in East Africa: Evidence from Demographic and health surveys of Ten East African countries

Manuscript Number: PONE-D-21-34591

**Background**

? Also in the introduction, I suggest that the study hypothesis be included.
The objective of the study may include the hypothesis that may considered because the study have two objectives (prevalence and factors of non-immunized rural women) then hypothesis is not that much important for this study due to the objective of the study.

? It is important for the authors to indicate why Uganda, a key East African country is not included in the list despite having a 2016 DHS

Ok, I have included it in the body of manuscript.

- ? 34,019 (1%) deaths from NT in 2015 as against 3.3 m annual ND cannot be described as a “high number” The authors may wish to rephrase the sentence to “despite progress with global efforts to eliminate MNT, 34,019 neonatal deaths were attributed to tetanus in 2015”

Ok, we accept it and we will try to correct the sentence.

? The statement “Maternal tetanus continues to be a major cause of neonatal and infant deaths in many developing countries” is not clear. I couldn't find such statements in the reference (Ref #6 & 7)

That is good, the reference can’t stat this sentence and we remove the phrase from the manuscript.

? Annual neonatal tetanus deaths were indicated for 2015 and 2017. The authors may wish to consider using the most recent estimates, which are 25,000 in 2018 (visit the link below).

We had correct it in the manuscript

? The statement “Similarly, the majority of the Sub-Saharan African countries could hardly reach the TT immunization target set to be covered [10]” requires that the authors indicate what the threshold TT coverage is referred to.

See the article (<https://doi.org/10.1186/s12914-018-0168-0>) in the background section.

? I was unable to find the statement “Somalia, South Sudan, Afghanistan, Kenya, Nigeria, and Ethiopia reported the highest rates of neonatal tetanus mortality (1,000 deaths per 100,000 population) [11]” in the cited reference. Please, ensure to quote relevant references to support the statement

Really, the reference doesn’t show clearly the above paragraph in Somalia, South Sudan, Afghanistan, Kenya, Nigeria, and Ethiopia but stated as the whole so we remove it from the manuscript section.

**Method**

? About the independent variables, how were they selected? Was it through a theoretical model?
The independent variable of the study was selected based on the literatures that were done in different time in different geographical location and also the variable obtained in the DHS data common in all selected countries in this study and categorized as the literature and the percentage of each category and their similarities.

? The variable income, as the classification "Poor, middle and Rich" was considered, I suggest that you make a brief description

The DHS data collected income as wealth index as Poorest, Poorer, Middle, Richer and Richest but based on their frequency and properties it is classified as Poorest and Poorer in to poor, middle in to middle and Richer and Richest in to rich for analysis to be simple and confortable.

? Still, in methods, I suggest including information about ethical aspects of the research.
Ok, we include in the main manuscript body.

1. The countries listed in this study Burundi, Kenya, Tanzania, Rwanda, Ethiopia, Comoros, Malawi, Zambia and Zimbabwe were found in East African countries (<https://doi.org/10.1186/s12889-020-09965-y>, Risk Management and Healthcare Policy 2020:13 1803–1813) compared with the whole Africa countries categorized as North, East, middle, west, south Africa. But which may be categorized as other sub geographical location depending on different cases. Comoros is located in the Indian Ocean at off coast of Eastern Africa. Comoros map shows the exact position of Comoros i.e. it is located in the northern side of Mozambique Channel and between Madagascar and Mozambique. Zimbabwe is situated in the southernmost part of East Africa.With Mozambique along the eastern. Border of Zimbabwe, the East African country of Zambia is to the north of Zimbabwe.
2. The percentage of TT immunization of women using graphs and percentages showed that the difference prevalence of TT+2 immunization between East Africa countries. The results of the study showed that the prevalence of ten east Africa countries and that showed that the different coverage of tetanus immunization of women’s. Therefore, this rural women’s tetanus immunization coverage was considered as compression based on coverage.
3. If the paper relied on secondary data analyses, which should be stated clearly, as it will appear that there, was extensive use of data already collected through the DHS.

Ok, we try to write the write the source of data again for more clarification for readers as much as possible, moreover, it is obtained online free link at (<https://www.dhsprogram.com/Data>).

4. The factor of TT protection of women was stated in the background of the study.

**Result**

? I suggest that in all tables, the sample number and year of data collection are included in the description.
Ok, we have correct it in the body of manuscript

? In table 4, include the meaning of *

We have removed it from the table because it is not that much important which is shown by P -Value level.

? Also in table 4, I suggest that a column with the value of p be included.

Ok, we have added it in the body of manuscript

1. ? It is not clear what the cut-off TT threshold coverage for protection is. Please state this

We try to justify it in the method section as women receive at least two TT2+ injections during pregnancy for her most recent birth, or two or more injections (the last within 3 years of the most recent birth), or three or more injections (the last within 5 years of the most recent birth), or four or more injections (the last within 10 years of the most recent birth), or five or more injections at any time before the most recent birth.

2. “Cameroon (50.3% not protected and 49.7% protected)” – Cameroon is not in East Africa and is not part of the list in the other sections of the manuscript. Please, crosscheck. Probably the authors meant Comoros.

Sorry it is writing error the perfect country name was Comoros

3. It is difficult to understand where percentage coverages quoted as “The prevalence of protected rural women from nine EA countries” come from. I looked up the TT2+ or Td2+ coverage figures reported through the most recent DHS reports in these countries and they do not seem to align with those reported in the manuscript. A clearer understanding in method section of how these figures were calculated would be useful.

It may be due to data extraction. This data can be extracted using SPSS software, first select the important variables for each country, delete the sample which is missing corresponding with the dependent variables for each country, finally merge each country data in one data set and recode some variables depending on some related literature.

4. ? From the method and result section, protection against tetanus is based on reported TT2+ or Td2+ coverage in the DHS. For several documented reasons, TT2+………

Really different scholars stated different methods but in this study we have use TT2 + to protect women from tetanus which is one type of tetanus redaction using TT2+ immunization.

5. In view of the wide disparities in health systems strength, demographics, development indices and economic status in the nine countries………….

The country have different performing strategy for elimination of tetanus but such listed countries may share the health and immunization policy for rural areas and have less urbanization peoples of most east Africa countries were live in rural areas with poor economic activates without enough infrastructure like health facilities and transportation. Therefore, we think that such listed countries may share the some status due to the above listed reasons.

6. were significantly correlated with full childhood immunization

it is typing error we have correct it in the manuscript.

Discussion

1. ? In the opening statement there seems to be an attempt to compare the findings from the nine “EA” countries lumped together with findings from single countries and even subnational levels of the countries.

That is, we have corrected it in the manuscript.

2. ? Reference is required for the statement “This might be the target population of this study

We cannot find reference but we have use the sentence as justification of the significance of women at first birth which may or may not true.

**Conclusions and recommendations**

1. **?** There appears to be many typos rendering the section unclear. The authors need to address these

We have made a change for correction

2. ? The sweeping conclusion about TT protection and the influencing factors in EA may make it difficult for readers to understand the extent of the problem at individual country level.

We had accepted it, and we will try to show the tetanus women’s immunization coverage of ten east Africa countries clearly.

**Limitations**

? There is no section that highlights the limitation of this study. The authors need to highlight the limitations

Ok, we had included in the manuscript section.
